# Pathologic Exhibit of the American Medical Association

**Published:** 1902-05

**Authors:** F. M. Jeffries, W. A. Evans, Roger G. Perkins

**Affiliations:** 214 E. 34th St., New York City. Committee on Pathologic Exhibit, American Medical Association. New York; 103 State St., Suite 1403, Chicago, Ill. Committee on Pathologic Exhibit, American Medical Association. New York; West. Res. Med. School, Cleveland, O. Committee on Pathologic Exhibit, American Medical Association. New York


### CORRESPONDENCE.

### Pathologic Exhibit of the American Medical Association.

*Editor Buffalo Medical Journal :*

*Sir.*—The committee on pathologic exhibit for the American Medical Association is anxious to secure materials for the coming session at Saratoga, June 10-13, 1902.

This exhibit was accorded much praise and comment during the sessions at Atlantic City and St. Paul respectively, where were collected valuable exhibits from all parts of the country. The materials included not only pathologic specimens but the allied fields, bacteriology, hematology, physiology and biology, were well represented. It would also be desirable to secure exhibits of new apparatus, charts, and the like, used by teachers of pathology and physiology in medical colleges.

This exhibit has already become a permanent feature of the annual sessions of the American Medical Association, hence the committee is desirous of securing its list of exhibits as early as possible and to this end asks those having desirable materials to communicate with any member of the committee.

To contribute to the value of the work, it is suggested that as far as possible each contributor select materials illustrative of one classification and by such specialisation enhance the usefulness of the display.

Those lending their materials may feel assured that good care will be given their exhibits while in the hands of the committee and due credit will be given in the published reports.

F. M. JEFFRIES,
214 E. 34th St., New York City.

W. A. EVANS,
103 State St., Suite 1403, Chicago, Ill.

ROGER G. PERKINS,
West. Res. Med. School, Cleveland, O.

Committee on Pathologic Exhibit, American Medical Association.

NEW YORK, March 26, 1902.

